# Does the lung cancer screening (LCS) pathway independently improve surgical outcomes compared with the routine pathway: a matched-case analysis

**DOI:** 10.1186/s13019-026-04023-x

**Published:** 2026-05-22

**Authors:** Ananya Mathur, Matar Alzahrani, Ambreen Abid, Madava Djearaman, Patricia Glynn, Nadeem Maddekar, Babu Naidu

**Affiliations:** 1 Birmingham Medical School, University of Birmingham, Birmingham, UK; 2 Institution of Inflammation and Aging, University of Birmingham, Birmingham, UK; 3 Queen Elizabeth Hospital Birmingham, Birmingham, UK; 4 King Abdullah International Medical Research Center, Jeddah, Saudi Arabia; 5 College of Applied Medical Sciences, King Saud Bin Abdulaziz University for Health Sciences, Jeddah, Saudi Arabia

**Keywords:** Lung Cancer Screening, Thoracic Surgery, 30 Day Mortality, Postoperative Complications, Length of Stay, Propensity Score Analysis, Matched-Case Analysis, Targeted Lung Health Check

## Abstract

**Background:**

Multiple randomised controlled trials demonstrate that screening is a game changer for lung cancer outcomes. As pilot Lung Cancer Screening schemes expand across the UK, we investigate whether referral through a screening pathway independently influences early postoperative outcomes following lung cancer resection, or whether observed benefits are due to differences in patient characteristics between screened and non-screened populations.

**Methods:**

A retrospective cohort analysis was conducted on 370 patients who underwent curative lung cancer resection. Of these, 35 were from the Lung Cancer Screening pathway, while 335 were diagnosed from standard of care pathways. Propensity Score Analysis was then used to create a matched cohort of 34 patients per group to minimise confounding baseline factors such as clinical characteristics and patient demographics. Postoperative outcomes including complications, length of stay, and 30-day mortality were then compared using univariate logistic and linear regression models. Imputation was used for any missing data.

**Results:**

Prior to matching, patients diagnosed via the Lung Cancer Screening pathway demonstrated more favourable baseline characteristics, subsequently requiring matching. Following matching, postoperative outcomes including length of stay (mean difference 1.81 days, p-value = 0.51), overall postoperative complication rate (OR -1.126, CI 0.701, 5.672, p-value = 0.195), complication severity objectively measured by Clavien-Dindo Classification (OR 2.039, CI 0.737, 5.638, p-value = 0.17), and Comprehensive Complication Index (OR 1.797, CI 0.644, 5.011, p-value = 0.263) were not statistically significantly different between the screened and non-screened populations. The 30-day mortality was 0% in the Lung Cancer Screening group, as opposed to 3.9% in the non-Lung Cancer Screening cohort (Absolute Risk Difference = 3.9%), however regression analysis could not be performed due to zero events in the screened group.

**Conclusions:**

The Lung Cancer Screening pathway appears to be indirectly associated with improved postoperative outcomes through patient selection rather than a direct, independent effect of the screening pathway. Larger studies are needed to validate these findings and elucidate how the downstream benefits of screening can be optimised across thoracic surgery pathways for lung cancer.

## Background

Lung cancer is the leading cause of cancer-related deaths worldwide and in the UK. Between 2017 and 2019, 49,229 cases of lung cancer were reported in the UK, with 34,771 cases resulting in deaths [1]. The high mortality rate can be attributed to late-stage diagnoses, often stemming from the absence of symptoms at earlier stages. As a result, patients present at an older age with multiple comorbidities, which makes treatment complex and contributes to higher mortality [2, 3].

These challenges underscore the urgent need for effective public health measures for early detection of lung cancer- most notably, lung cancer screening, which has strongly demonstrated the reduction of mortality through major trials such as NLST [4], NELSON [5], and UKLS [6]. Within the UK, one such key initiative is the implementation of the Lung Cancer Screening (LCS) programme, which is being nationally rolled out in the UK based on the success of its pilot programme.

The LCS programme, previously referred to as the Targeted Lung Health Check (TLHC) [7], aims to identify lung cancer in ever-smokers aged 55–74 registered with a General Practitioner [8]. These individuals undergo risk-assessment using tools such as the PLCOm2012 risk calculator [9] and Liverpool Lung Project model [10]. Those classified as high risk are subsequently invited for Low Dose CT (LDCT) screening conducted in community settings. This risk-stratified approach facilitates early lung cancer detection by targeting individuals with the highest predisposition to developing cancer, thereby allowing for potentially curative surgical resection.

Minimally invasive lung cancer resection is considered the gold standard for early-stage lung cancer [11], as it is associated with reduced postoperative pain, shorter hospital stays, better recovery, and fewer complications [12]. While lung cancer screening is clearly associated with improved surgical outcomes, it is uncertain whether better surgical outcomes in patients undergoing lung cancer screening are due to the screening pathway itself, or instead are impacted by confounding factors like tumour biology, health-seeking behaviour, or socioeconomic status. Therefore, we propose conducting a matched case analysis of patients receiving curative lung cancer surgical resection, comparing those referred through the LCS pathway with those via the conventional lung cancer pathway at a single tertiary referral unit, to identify if screening is an independent prognostic factor of early postoperative clinical outcomes.

## Methods

### Study design

This is a cohort study that included a retrospective analysis of a prospectively collected database at a single centre, followed by a matched case analysis to compare outcomes between patients referred via the Lung Cancer Screening pathway and those diagnosed through conventional pathways. This comparison was then used to evaluate whether differences in early postoperative outcomes were independently associated with screening referral, rather than attributable to confounding factors.

### Participants and procedures

Data was collected from a pre-existing, prospectively maintained electronic database of patients who underwent thoracic surgery for primary lung cancer at Queen Elizabeth Hospital Birmingham between December 2022 and September 2024 (*n* = 370).

From this database, patients were classified into two groups based on their diagnostic pathway. The LCS group (*n* = 35) included individuals identified through the LCS programme, who were ever-smokers aged 55–74. In contrast, the non-LCS group (*n* = 335) comprised individuals diagnosed through the standard of care pathway, with no restrictions on age or smoking history.

After patients were classified into LCS and non-LCS groups, baseline characteristics and postoperative outcomes were collected.

### Baseline characteristics

Baseline characteristics that were taken into consideration for both groups were age at surgery, sex, smoking status, pre-existing comorbidities, preoperative lung function tests [Forced Expiratory Volume % (FEV%), Forced Vital Capacity % (FVC%), Transfer Factor for Carbon Monoxide (TLCO)], tumour staging [Tumour, Node, and Metastasis (TNM)], type of surgical intervention (Pneumonectomy, Lobectomy, segmental resection), and type of surgical approach [Thoracotomy, Video-Assisted Thoracoscopic Surgery (VATS), Robotic-Assisted Thoracoscopic Surgery (RATS)].

### Outcome measures

The primary outcomes measured were postoperative length of stay (PLOS), 30-Day Mortality (30DM), and postoperative complications (POC). These outcomes were extracted from patients’ electronic medical records.

PLOS was evaluated as an indicator of the efficiency of perioperative care and postoperative healthcare resource utilisation [13].

30DM was measured as another key outcome as it is an established benchmark for postoperative mortality in thoracic surgery [14] and is used in the Society of Thoracic Surgeons (STS) National Database [15]. This ensures standardisation and comparability of our outcomes across other studies and between LCS and non-LCS groups.

POC were evaluated too as they reflect morbidity and mortality post-surgery, alongside a patient’s recovery process [16]. In order to assess POC objectively, all complications were classified into different grades as per the Clavien-Dindo Classification (CDC) [17]. These grades were inputted into the Comprehensive Complication Index (CCI^®^) Calculator [18], which generated a final overall numerical score, with higher values indicating more severe complications [19].

### Imputation and logistic regression

Once baseline characteristics and postoperative outcomes were collected, missing baseline data in the non-LCS group was imputed using the Multivariate Imputation by Chained Equations (MICE) algorithm in R [20].

Next, to determine whether patients referred via the LCS and non-LCS pathways had comparable baseline characteristics prior to outcome comparison, a univariate logistic regression analysis was performed. This generated odds ratios (ORs) which described the association between individual baseline characteristics and LCS referral status, thereby identifying whether systematic differences existed between cohorts [21]. The presence of such associations indicated baseline imbalance and potential confounding that could bias comparisons of postoperative outcomes. To address this and improve the validity of between-group comparisons, a matched case analysis was subsequently performed to enhance comparability between cohorts and minimise selection bias. A 95% Confidence Interval (CI) was chosen during this analysis, as this is the standard in medical literature [22].

### Matching, covariates, and statistical analysis

Propensity Score Analysis (PSA) was conducted using the MatchIt package in R [23] to match patients between cohorts. Within this analysis, propensity scores were derived from a multivariable logistic regression model incorporating baseline characteristics. Based on these scores, 1:1 matching of 34 patients from each group was performed.

A nearest-neighbour matching was employed, where patients who were most alike from both the groups were found. A calliper of 0.02 was utilized, which meant that the difference in propensity scores of the two groups was required to be within a margin of ± 0.02 for matching to happen. This was chosen to ensure that matches remained extremely similar, while also ensuring that there were not too few matches. In addition, the matching was 1:1 without replacement, meaning that for every patient from the non-LCS group, we had a match with exactly one patient from the LCS group. Of the 35 patients initially identified in the LCS cohort, one patient could not be matched within the specified calliper and was therefore excluded, resulting in a final matched cohort of 34 patients in each group.

**Fig. 1:**
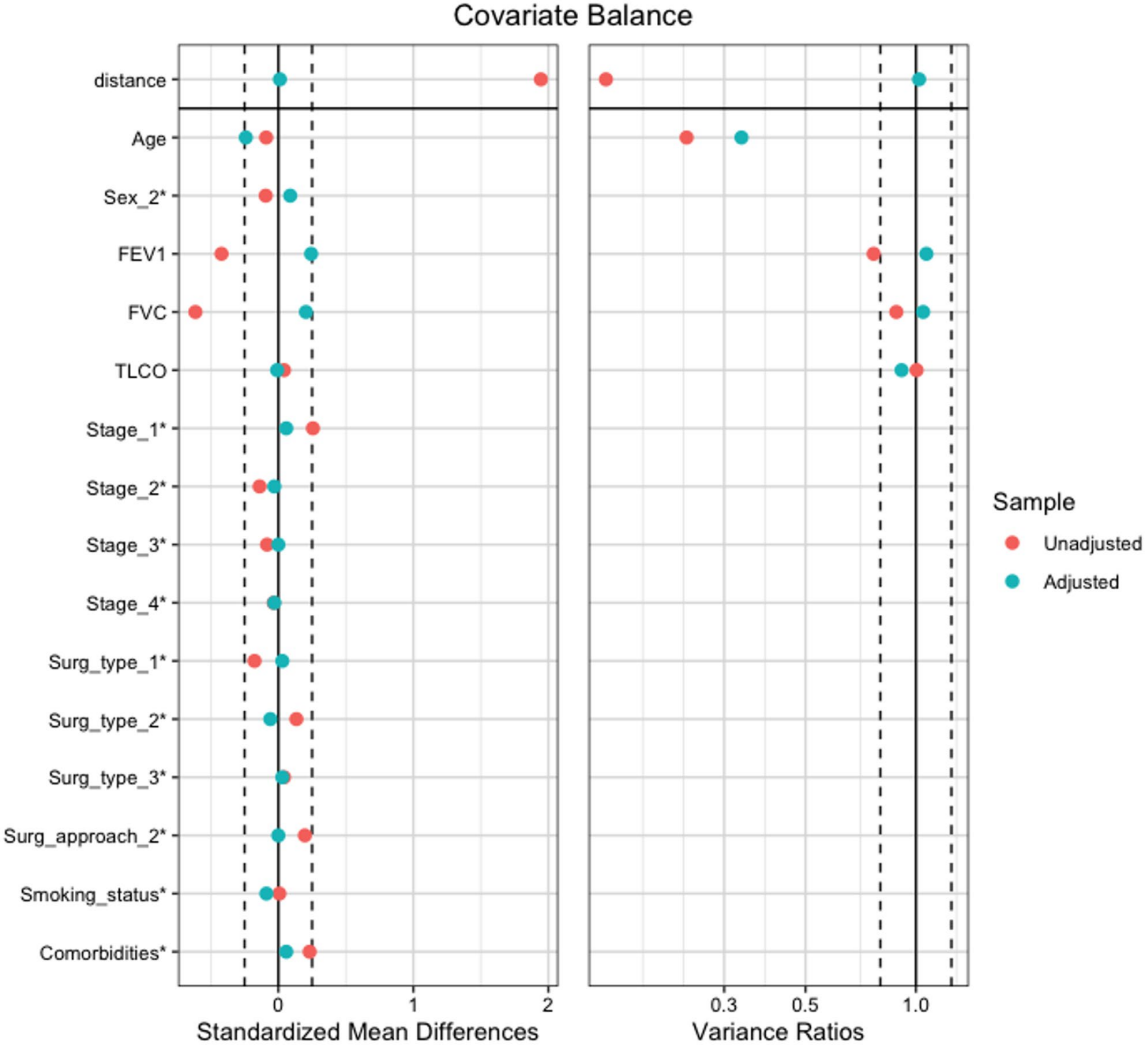
Covariates balance assessment before and after propensity score matching

After carrying out matching, we verified the comparability of the two groups by checking the balance of covariates. Once this was done, we computed the effect estimate and standard errors with the match population. Figure 1 shows the standardised mean differences for covariates before (red) and after (blue) adjustment in PSA. The two vertical dashed lines represent the thresholds for acceptable balance, typically set at ± 0.1, indicating covariates with differences within these lines are considered well-balanced (Fig. 1).

### Comparing outcomes

Linear regression was used to analyse mean differences in PLOS between cohorts, as this is a continuous variable. Categorical postoperative outcomes, including complications, CCI, and CDC, were analysed using logistic regression. These statistical analyses were performed in order to quantify differences in postoperative outcomes and to assess their effect estimates alongside statistical significance, rather than relying on descriptive comparison.

## Results

### General patient characteristics

**Fig. 2:**
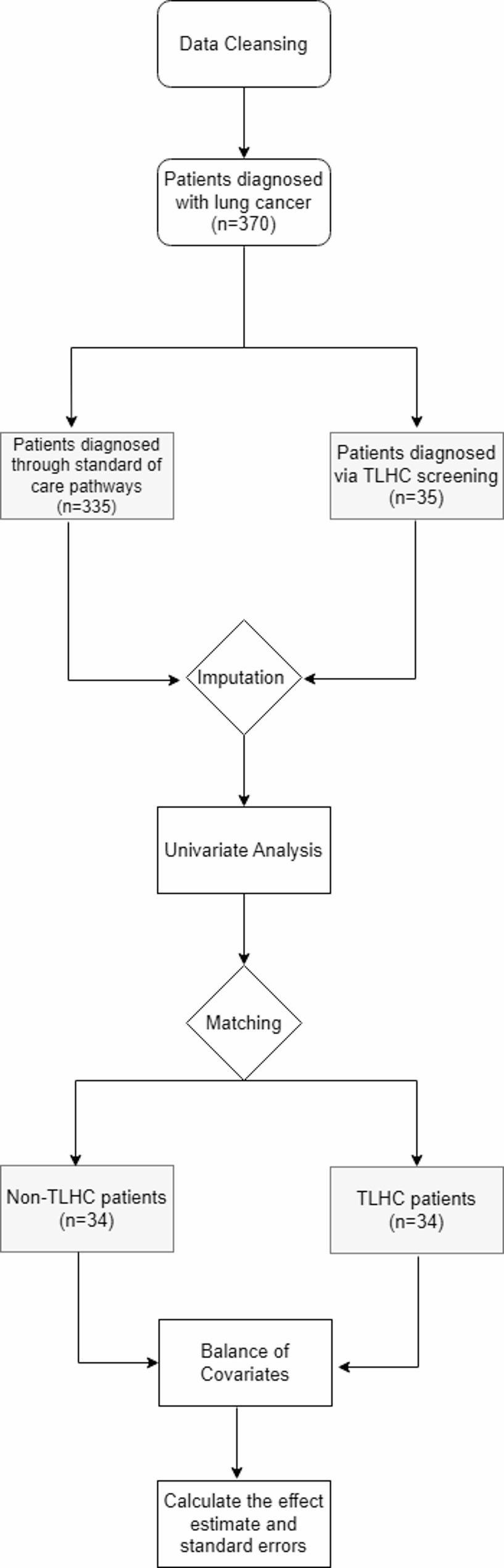
PSA steps flowchart for evaluating impact of LCS on lung cancer surgery patients

A total of 370 LCS and non-LCS patients who underwent thoracic surgery for primary lung cancer between 2022 and 2024 were included in the statistical analysis (Fig. 2). Baseline characteristics and postoperative outcomes of both groups prior to matching are reported in Table 1 as observed, with missing data included. The non-LCS matched cohort with changed baseline characteristics and outcomes is included to illustrate how propensity score matching improved comparability with the LCS group.

**Table 1:** Baseline characteristics and outcomes of 370 patients included in the study

Characteristic	Non-LCS unmatched cohort (*n* = 335)	LCS unmatched cohort (*n* = 35)	Non-LCS matched cohort (*n* = 34)
**Patient demographics**			
Age (Years)	70 (62, 75)	69 (64, 71)	68 (62, 75)
Sex (Male)	141 (42%)	18 (51%)	17 (50%)
Sex (Female)	194 (58%)	17 (49%)	17 (50%)
**Lung Function**			
% FEV_1_	87 (73, 101)	81 (66, 91)	76 (64.5, 89.5)
% FVC	99 (88, 109)	86 (78, 98)	89 (77.5, 101)
% predicted TLCO	86 (71, 99)	91 (72, 100)	89 (81.5, 107)
**Smoking Status**			
Non-Smoker	47 (18%)	6 (17%)	6 (18%)
Smoker	213 (82%)	29 (83%)	28 (82%)
**Comorbidities**			
Absent	116 (35%)	4 (11%)	5 (15%)
Present	219 (65%)	31 (89%)	29 (85%)
**Cancer Stage**			
1	198 (69%)	33 (94%)	26 (76%)
2	48 (17%)	1 (2.9%)	5 (14.7%)
3	26 (9.0%)	0 (0%)	2 (5.9%)
4	17 (5.9%)	1 (2.9%)	1 (2.9%)
**Surgery Type**			
Lobectomy	260 (78%)	21 (60%)	22 (65%)
Wedge	70 (21%)	12 (34%)	10 (29%)
Segmentectomy	5 (1.5%)	3 (5.7%)	2 (5.9%)
**Surgical approach**			
Thoracotomy	84 (26%)	2 (5.7%)	6 (18%)
RATS/VATS	245 (74%)	33 (94%)	28 (82%)
**PLOS**	5.0 (3.0, 8.0)	5.0 (5.0, 6.5)	5.0 (4.0, 8.0)
**DM30**			
Survived	255 (98%)	35 (100%)	33 (97%)
Died	5 (1.9%)	0 (0%)	1 (2.9%)
**POC**			
No Complications	27 (77%)	22 (63%)	26 (76%)
Complications	8 (23%)	13 (37%)	8 (24%)
**CDC Class**			
0	27 (77%)	21 (60%)	26 (76%)
1	1 (2.9%)	4 (11%)	1 (2.9%)
2	3 (8.6%)	5 (14%)	3 (8.8%)
3	3 (8.6%)	2 (5.7%)	2 (5.9%)
4	0 (0%)	2 (5.7%)	1 (2.9%)
5	1 (2.9%)	1 (2.9%)	1 (2.9%)
**CCI**			
0	27 (77%)	21 (60%)	26 (76%)
8.7	1 (2.9%)	4 (11%)	1 (2.9%)
20.9	3 (8.6%)	5 (14%)	3 (8.8%)
26.2	3 (8.6%)	2 (5.7%)	2 (5.9%)
33.7	0 (0%)	2 (5.7%)	1 (2.9%)
42.4	1 (2.9%)	1 (2.9%)	1 (2.9%)

Data is presented as mean (median), standard deviation (interquartile range), or numbers and percentages for categorical data. LCS; Lung Cancer Screening, FEV_1_; Forced Expiratory Volume for 1 s, FVC; Forced Vital Capacity, TLCO; Transfer Factor for Carbon Monoxide VATS; Video-Assisted Thoracic Surgery, RATS; Robotic-Assisted Thoracic Surgery

The median age is similar between the LCS and non-LCS unmatched cohorts, with a higher proportion of females observed in the non-LCS population. Preoperative lung function tests suggest an inconclusive pattern among the two groups. Smoking status is nearly identical between the groups, while comorbidities are interestingly higher in the LCS population. LCS patients tend to present at earlier stages, with none at stage 3 and only one at stage 4. Additionally, they are more likely to undergo minimally invasive surgeries likes VATS/RATS and wedge resections compared to non-LCS patients (Table 1).

These baseline differences were more closely balanced following matching, with baseline characteristics appearing more comparable between the LCS unmatched and non-LCS matched cohort columns of Table 1.

### Univariate logistic regression analysis results

Table 2 shows the results of univariate logistic regression analysis that demonstrated baseline associations with LCS status, and this motivated the subsequent match process. The analysis demonstrated that several baseline factors did not significantly influence whether a patient was in the LCS group. Age, sex, preoperative lung function tests, and smoking status had ORs close to 1, indicating no meaningful association. However, comorbidities were statistically significantly associated with LCS status, with patients with more comorbidities having increased odds of being in the LCS group.

For cancer stage, patients with stage 1 disease had lower odds of being in the non-LCS group (OR = 0.12, CI 0.01, 0.60) compared to stage 2 patients, meaning stage 1 patients were significantly more likely to be LCS compared to stage 2 patients. A similar trend was seen for stage 4 patients, reinforcing that LCS patients tend to present at earlier stages. In terms of surgical approach, LCS patients had higher odds of undergoing minimally invasive procedures (RATS/VATS), compared to thoracotomy (OR = 5.61, CI 1.66, 35.1). Similarly, wedge resections (OR = 2.12, CI 0.97, 4.46) and segmentectomies (OR = 4.19, CI 0.68, 24.6) were significantly more likely in LCS patients compared to lobectomies.

Postoperatively, LCS patients were less likely to experience complications than non-LCS patients. The odds of having a lower CDC class were higher in LCS patients, but confidence intervals crossed 1, making definitive conclusions difficult. CCI and PLOS were not significantly different between the two groups (OR ≈ 1). There was no mortality observed in the LCS group.

Since there were no LCS patients in Stage 3, logistic regression could not be performed for this variable.

**Table 2:** Results of univariate logistic regression analysis on 370 patients

Characteristic	Non-LCS [*n* = 335]	LCS [*n* = 35]	OR	95% CI	*p*-value
**Age** (Years)	70 (60, 75)	69 (64, 71)	1.00	0.96, 1.03	0.80
**Sex**					
Male	141 (42%)	18 (51%)	Reference	-	-
Female	194 (58%)	17 (49%)	0.69	0.34, 1.38	0.29
**% FEV1**	87 (73, 101)	81 (66, 91)	0.98	0.96, 1.00	0.035
**% FVC**	99 (88, 110)	86 (78, 98)	0.97	0.95, 0.99	0.001
**TLCO**	86 (71, 100)	91 (72, 100)	1.00	0.99, 1.02	0.81
**Smoking Status**					
Non-Smoker	60 (18%)	6 (17%)	Reference	-	-
Smoker	275 (82%)	29 (83%)	1.05	0.45, 2.91	0.91
**Comorbidities**					
Absent	116 (35%)	4 (11%)	Reference	-	-
Present	219 (65%)	31 (89%)	4.11	1.58, 14.0	0.002
**Cancer Stage**					
1	230 (69%)	33 (94%)	Reference	-	-
2	56 (17%)	1 (2.9%)	0.12	0.01, 0.60	0.002
3	28 (8.4)	0 (0%)	0.00	-	-
4	21 (6.3%)	1 (2.9%)	0.33	0.02, 1.67	-
**Surgery Type**					0.059
Lobectomy	260 (78%)	21 (60%)	Reference	-	-
Wedge	70 (21%)	12 (34%)	2.12	0.97, 4.46	-
Segmentectomy	5 (1.5%)	2 (5.7%)	4.95	0.68, 24.6	-
**Surgical approach**					
Thoracotomy	85 (25%)	2 (5.7%)	Reference	-	-
RATS/VATS	250 (75%)	33 (94%)	5.61	1.66, 35.1	0.003

OR; Odds Ratio, CI; Confidence Interval

LCS; Lung Cancer Screening, FEV_1_; Forced Expiratory Volume for 1 s, FVC; Forced Vital Capacity, TLCO; Transfer Factor for Carbon Monoxide VATS; Video-Assisted Thoracic Surgery, RATS; Robotic-Assisted Thoracic Surgery

### Patient characteristics after matching

Since we were able to deduce a correlation between certain baselines and outcomes, propensity score matching was performed. The effect mean differences, along with standard errors, were calculated for post-surgical outcomes, including PLOS, POC, CDC class, and CCI. Propensity score analysis for 30-day mortality was not possible because there were no deaths in the LCS group. With zero events in one group, there is no variability in the outcome to model or compare, making it statistically invalid to estimate treatment effects.

### Outcome 1 - postoperative length of stay

A univariate linear regression analysis demonstrated no statistically significant association between LCS status and postoperative length of stay in the matched cohort. Although patients diagnosed via the LCS pathway had a numerically shorter postoperative stay compared with non-LCS patients (mean difference of -1.8 days), this difference was not statistically significant (*p* = 0.51) (Table 3).

**Table 3:** Results of univariate linear regression analysis against postoperative length of stay

Variable	Mean Difference	Standard Error	t-value	*p*-value
**LCS Status** (vs. non-LCS)	-1.80975	2.75985	-0.656	0.5148

LCS; Lung Cancer Screening

### Outcome 2 - postoperative complications

Univariate logistic regression analysis demonstrated no statistically significant association between LCS status and the occurrence of postoperative complications in the matched cohort (p-value − 0.20) (Table 4).

**Table 4:** Results of logistic regression analysis against postoperative complications

Variable	Odds Ratio (OR)	Confidence Interval (CI)	*p*-value
**LCS Status** (vs. non-LCS)	-1.126	0.701, 5.672	0.195533

LCS; Lung Cancer Screening

#### CDC class

Ordinal logistic regression analysis demonstrated no statistically significant association between LCS status and Clavien–Dindo complication grade in the matched cohort (p value − 0.17) (Table 5).

**Table 5:** Results of logistic regression analysis against clavien-dindo classification

Variable	Odds Ratio (OR)	Confidence Interval (CI)	*p*-value
**LCS Status** (vs. non-LCS)	2.039	0.737, 5.638	0.1700

LCS; Lung Cancer Screening

#### CCI

Ordinal logistic regression analysis demonstrated no statistically significant association between LCS referral status and Comprehensive Comorbidity Index category in the matched cohort (p value − 0.26) (Table 6).

**Table 6:** Results of logistic regression analysis against comprehensive complication index

Variable	Odds Ratio (OR)	Confidence Interval (CI)	*p*-value
**LCS Status** (vs. non-LCS)	1.797	0.644, 5.011	0.2627

LCS; Lung Cancer Screening

### Outcome 3–30 day mortality

As indicated previously, this analysis could not be conducted for 30 Day Mortality as LCS patients had a 0% 30DM, as opposed to the non-LCS group with a 3.9% mortality rate.

## Discussion

The real-world effectiveness of Lung Cancer Screening (LCS) in improving lung cancer outcomes post thoracic surgery remains underexplored. While international landmark studies like the NELSON and NLST trials have emphasised the value of low-dose CT screening in reducing mortality and UK based literature on LCS primarily investigates diagnostic outcomes, evidence on surgical outcomes and their downstream impact is lacking. This study aimed to address this gap by evaluating surgical outcomes in a matched cohort of LCS-diagnosed patients versus those diagnosed through standard of care pathways, specifically identifying whether any observed benefits are independently attributable to screening itself or instead reflect differences in the population identified by screening.

In the unmatched cohort, patients through the LCS pathway demonstrated more favourable baseline characteristics compared with those diagnosed through conventional pathways. These included earlier stage disease, higher rates of minimally invasive surgical approaches and more limited resections. These characteristics have been previously associated with improved postoperative outcomes, including shorter postoperative hospital stays, reduced mortality, and lower complication rates. Univariate logistic regression further confirmed that several of these favourable baseline characteristics were significantly associated with LCS referral status, consistent with previously published screening literature. These findings indicated that patients entering from the LCS pathway differed systematically from the non-LCS pathway, suggesting that the two groups were not directly comparable at baseline without the risk of confounding.

The presence of these imbalances led to the use of propensity score matching to create matched cohorts and remove any association between baseline characteristics and postoperative outcomes. When postoperative outcomes were analysed within this matched population, differences in outcomes were attenuated and no longer statistically significant. Postoperative length of stay and complication rates, quantified by the Clavien-Dindo classification and Comprehensive Complication index, did not differ significantly between the LCS and non-LCS groups (all *p* > 0.05). Although some outcomes continued to show numerical trends favouring the LCS cohort, confidence intervals were wide and crossed null-values, indicating uncertainty around these effect estimates.

The attenuation of outcome differences after matching suggests that the favourable postoperative outcomes observed in the unmatched LCS cohort were largely explained by the baseline differences, rather than by the screening pathway itself exerting a direct independent effect on postoperative surgical recovery. Hence, lung cancer screening appears to improve surgical outcomes by identifying patients with more favourable disease characteristics - such as earlier-stage tumours amenable to minimally invasive and less extensive surgery- rather than through an intrinsic effect of the screening pathway itself on postoperative outcomes.

These findings revalidate existing literature on screening. Screening seems to identify patients with a lower disease burden, which then increases the likelihood of undergoing minimally invasive surgery, which in turn leads to improved postoperative outcomes. This suggests that once these baseline factors are accounted for, any additional, unmeasured pathway-specific advantages – such as earlier or multiple multidisciplinary team (MDT) discussions, more planned surgical scheduling, faster preoperative work-up, or patient behavioural differences in seeking treatment - do not contribute to improved postoperative outcomes. This provides further reassurance that observed surgical benefits associated with LCS are primarily achieved through patient selection rather than other confounding healthcare delivery factors that may be associated with screening.

Given this, some limitations should be acknowledged. As a retrospective, observational study, it was prone to missing data and potential unmeasured confounding factors. However, this was tackled through data imputation and robust statistical methods such as propensity score matching which enhanced the internal validity of the results. The small, matched sample size (*n* = 34) has limited statistical power, increasing the risk of type II error. Additionally, being a single-centre study, the results may not be fully generalisable to all healthcare settings within the UK. That being said, the hospital at which data collection was carried out serves a large and socioeconomically diverse catchment area of approximately 6 million patients. Thus, these findings may still remain relevant and potentially transferrable to the target population, while also serving as a foundation for future multi-centre, larger cohort studies.

Further multicentre studies with larger screened cohorts are required to validate these findings, explore more longer-term surgical outcomes, and better understand which characteristics of screening-detected patients contribute most to improved postoperative outcomes. This may help guide efforts strategies to extend the benefits of early diagnosis and favourable surgical characteristics to patients diagnosed outside screening pathways as well.

## Conclusion

Patients diagnosed through Lung Cancer Screening (LCS) have been shown to experience more favourable postoperative outcomes following lung cancer surgery. In this matched cohort study, it was demonstrated that the LCS pathway was not independently associated with improved early postoperative outcomes following thoracic surgery. After adjusting for different baseline characteristics between screened and non-screened patients, postoperative outcomes including length of stay, complication rates and complication severity were comparable between groups. These findings demonstrate that improved outcomes in screened patients are likely due to patient selection in screened populations rather than a direct effect of the screening pathway itself. Lung cancer screening remains a crucial public health intervention for reducing lung cancer mortality, and hence further larger scale studies are required to better understand how which components of the screening pathway most influence surgical outcomes to optimise these pathways better.

## Data Availability

The data that support the findings of this study are available from Queen Elizabeth Hospital Birmingham but restrictions apply to the availability of these data, which were used under license for the current study, and so are not publicly available. Data are however available from the authors upon reasonable request and with permission of Queen Elizabeth Hospital Birmingham.

## Abbreviations

LCS: Lung cancer screening
TLHC: Targeted lung health check
NLST: National lung screening trial
NELSON: Nederlands–Leuvens Longkanker Screenings ONderzoek
UKLS: United Kingdom lung cancer screening trial
LCS: Lung cancer screening
LDCT: Low dose computed tomography
FEV: Forced expiratory volume
FVC: Forced vital capacity
TLCO: Transfer factor for carbon monoxide
TNM: Tumour, node, and metastasis
VATS: Video-assisted thoracoscopic surgery
RATS: Robotic-assisted thoracoscopic surgery
PLOS: Postoperative length of stay
30DM: 30 day mortality
POC: Postoperative complications
STS: Society of thoracic surgeons
CDC: Clavien-Dindo classification
CCI: Comprehensive complication index
MICE: Multivariate imputation by chained equations
CI: Confidence interval
OR: Odds ratio
MDT: Multidisciplinary team

## References

[1] Cancer Research UK. April. https://www.cancerresearchuk.org/health-professional/cancer-statistics/statistics-by-cancer-type/lung-cancer. Accessed 5 2025.

[2] Harker R. House of Commons Library. https://researchbriefings.files.parliament.uk/documents/SN06887/SN06887.pdf. Accessed 13 April 2025.

[3] Schabath MB, Cote ML. Cancer progress and priorities: lung cancer. Cancer Epidemiol Biomarkers Prev. 2019; 1;28(10):1563–79. 10.1158/1055-9965.EPI-19-0221 PMC6777859 31575553

[4] The National Lung Screening Trial Research Team. Reduced Lung-Cancer Mortality with Low-Dose Computed Tomographic Screening. NEJM. 2011; 4;365(5):395–409. 10.1056/NEJMoa1102873 PMC4356534 21714641

[5] Ru Zhao Y, Xie X, de Koning HJ, Mali WP, Vliegenthart R, Oudkerk M. NELSON lung cancer screening study. Cancer Imaging. 2011;11(1A):S79–84. 22185865 10.1102/1470-7330.2011.9020 PMC3266562

[6] Field JK, Vulkan D, Davies MPA, Baldwin DR, Brain KE, Devaraj A, et al. Lung Cancer Mortality Reduction by LDCT screening: UKLS Randomised Trial Results and International meta-analysis. Lancet Reg Health Eur. 2021;10(100179):100179. 34806061 10.1016/j.lanepe.2021.100179 PMC8589726

[7] UK National Screening Committee. https://nationalscreening.blog.gov.uk/2025/01/31/targeted-lung-health-check-programme-renamed-the-nhs-lung-cancer-screening-programme/. Accessed 13 April 2025.

[8] NHS England. https://www.england.nhs.uk/wp-content/uploads/2019/02/B1646-standard-protocol-targeted-lung-health-checks-programme-v2.pdf. Accessed 30 March 2025.

[9] Tammemägi MC, Katki HA, Hocking WG, Church TR, Caporaso N, Kvale PA, et al. Selection Criteria Lung-Cancer Screen NEJM. 2013;368(8):728–36. 23425165 10.1056/NEJMoa1211776 PMC3929969

[10] Marcus MW, Chen Y, Raji OY, Duffy SW, Field JK. LLPi: Liverpool Lung Project Risk Prediction Model for Lung Cancer Incidence. Cancer Prev Res. 2015;8(6):570–5. 10.1158/1940-6207.CAPR-14-0438 25873368

[11] Hartwig MG, D’Amico TA. Thoracoscopic Lobectomy: The Gold Standard for Early-Stage Lung Cancer? Ann Thorac Surg. 2010;89(6):S2098–101. 20493989 10.1016/j.athoracsur.2010.02.102

[12] Lim E, Batchelor TJP, Dunning J, Shackcloth M, Anikin V, Naidu B et al. Video-Assisted Thoracoscopic or Open Lobectomy in Early-Stage Lung Cancer. NEJM Evid. 2022;1(3). 10.1056/EVIDoa2100016 10.1056/EVIDoa2100016 38319202

[13] Giambrone GP, Smith MR, Wu X, Gaber-Baylis LK, Bhat AU, Ramin Zabih, et al. Variability in length of stay after uncomplicated pulmonary lobectomy: is length of stay a quality metric or a patient metric? Eur J Cardiothorac Surg. 2016;49(4):e65–71. 26823164 10.1093/ejcts/ezv476 PMC5006293

[14] McMillan R, Berger A, Sima CS, Lou F, Dycoco J, Rusch VW, et al. Thirty-Day Mortality Underestimates the Risk of Early Death After Major Resections for Thoracic Malignancies. Ann Thorac Surg. 2014;98(5):1769–75. 25200731 10.1016/j.athoracsur.2014.06.024 PMC4410352

[15] Mortality Status Fields. STS. 2025. https://www.sts.org/registries-research-center/sts-national-database/mortality-status-fields. Accessed 18 April 2025.

[16] Tevis SE, Kennedy GD. Postoperative complications and implications on patient-centered outcomes. J Surg Res. 2013;181(1):106–13. 23465392 10.1016/j.jss.2013.01.032 PMC3637983

[17] Dindo D, Demartines N, Clavien PA. Classification of Surgical Complications. Ann Surg. 2004;240(2):205–13. 15273542 10.1097/01.sla.0000133083.54934.ae PMC1360123

[18] Clavien PA, Vetter D, Staiger RD, Slankamenac K, Mehra T, Graf R, et al. The Comprehensive Complication Index (CCI^®^). Ann Surg. 2017;265(6):1045–50. 28486288 10.1097/SLA.0000000000002132

[19] CCI^®^ Calculator. https://cci-calculator.com/cci-calculator‌. Accessed 19 April 2025.

[20] van Ravenzwaaij D, Cassey P, Brown SD. A simple introduction to Markov Chain Monte–Carlo sampling. Psychon Bull Rev. 2016;25(1):143–54. 10.3758/s13423-016-1015-8 PMC5862921 26968853

[21] Szumilas M. Explaining Odds Ratios. J Can Acad Child Adolesc Psychiatry. 2010;19(3):227. 20842279 PMC2938757

[22] Hazra A. Using the Confidence Interval Confidently. J Thorac Dis. 2017;9(10):4124–9. 10.21037/jtd.2017.09.14 PMC5723800 29268424

[23] Lee B, Kim N, Won S, Gim J. Propensity score matching for comparative studies: a tutorial with R and Rex. J Min Invasive Surg. 2024;27(2):55–71. 10.7602/jmis.2024.27.2.55 PMC11187614 38886996

